# Effectiveness of Antiviral Therapy on Long COVID: A Systematic Review and Meta-Analysis

**DOI:** 10.3390/jcm12237375

**Published:** 2023-11-28

**Authors:** Yu Jung Choi, Yu Bin Seo, Jun-Won Seo, Jacob Lee, Eliel Nham, Hye Seong, Jin Gu Yoon, Ji Yun Noh, Hee Jin Cheong, Woo Joo Kim, Eun Jung Kim, Joon Young Song

**Affiliations:** 1 Division of Infectious Diseases, Department of Internal Medicine, Korea University College of Medicine, Seoul 02841, Republic of Korea; yujungchoi@kumc.or.kr (Y.J.C.); neliel0106@gmail.com (E.N.); msmjoonhoo@gmail.com (H.S.); zephirisj9@gmail.com (J.G.Y.); lundisoir@hanmail.net (J.Y.N.); heejinmd@korea.ac.kr (H.J.C.); wjkim@korea.ac.kr (W.J.K.); 2 Vaccine Innovation Center-KU Medicine, Seoul 02841, Republic of Korea; 3 Division of Infectious Disease, Department of Internal Medicine, Kangnam Sacred Heart Hospital, Hallym University College of Medicine, Seoul 07442, Republic of Korea; yubinseo@gmail.com (Y.B.S.); litjacob@hallym.or.kr (J.L.); 4 Departments of Internal Medicine, College of Medicine, Chosun University, Gwangju 61452, Republic of Korea; kaist-105@hanmail.net; 5 Health, Welfare, Family and Gender Equality Team, National Assembly Research Service, Seoul 07233, Republic of Korea

**Keywords:** long COVID-19, antiviral therapy, SARS-CoV-2, systemic review, meta-analysis, post-acute sequelae

## Abstract

Antiviral treatment reduces the severity and mortality of SARS-CoV-2 infection; however, its effectiveness against long COVID-19 is unclear. This study aimed to evaluate the effectiveness of antiviral drugs in preventing long COVID and related hospitalizations/deaths. Scientific and medical databases were searched from 1 January 2020 to 30 June 2023. We included observational cohort studies comparing individuals receiving early antiviral therapy for COVID-19 and those receiving supportive treatment. A fixed-effects model was used to merge the effects reported in two or more studies. The risk of post-acute sequelae of COVID-19 (PASC) was combined as an odds ratio (OR). Six studies were selected, including a total of 3,352,235 participants. The occurrence of PASC was 27.5% lower in patients who received antiviral drugs during the early stages of SARS-CoV-2 infection (OR = 0.725; 95% confidence interval [CI] = 0.409–0.747) than in the supportive treatment group. Moreover, the risk of PASC-associated hospitalization and mortality was 29.7% lower in patients receiving early antiviral therapy than in the supportive treatment group (OR = 0.721; 95% CI = 0.697–0.794). Early antiviral therapy was associated with a reduced risk of PASC and related hospitalization or death. Thus, early antiviral therapy is recommended for at-risk individuals.

## 1. Introduction

Over the past 3 years, the emergence of severe acute respiratory syndrome coronavirus 2 (SARS-CoV-2) has triggered a global crisis. As of 26 July 2023, the World Health Organization reported approximately 768 million confirmed cases and 7 million deaths [1]. SARS-CoV-2 infection causes a wide spectrum of illnesses, including mild respiratory symptoms, atypical manifestations, and multiorgan dysfunction leading to fatal illness.

Even after recovery from acute SARS-CoV-2 infection, some patients suffer from long-lasting sequelae with diverse manifestations involving various organ systems [2,3,4]. General weakness, fatigue, dyspnea, myalgia/arthralgia, and psychological symptoms (depression, anxiety, sleep disturbance, and attention deficits) are common manifestations [2,3,5]. This long-lasting illness (more than 2–3 months), not explained by another diagnosis, has been referred to as long COVID, also known as post-acute sequelae of COVID-19 (PASC) [6]. More than 10% of survivors experienced long COVID, resulting in an increased medical cost burden [7].

Several studies have reported risk factors for long COVID [5,8,9,10]. According to a telephone interview survey of 274 individuals with COVID-19 in an outpatient setting, old age was the most contributing risk factor of long COVID, followed by the presence of hypertension, obesity, and psychiatric conditions [11]. Similarly, in another study, long COVID was significantly associated with age, increasing from 9.9% in individuals aged 18–49 years to 21.9% in those aged ≥ 70 years (*p* < 0.0005). Individuals of the female sex and those with underlying asthma have also been reported to be more susceptible to long COVID [8].

Antiviral treatment is effective in reducing hospitalization and death in patients with a SARS-CoV-2 infection [12]; however, whether antiviral treatment is beneficial in preventing long COVID is unclear. The aim of this study was to evaluate the effectiveness of antiviral agents in preventing long COVID using a systematic review and meta-analysis, thereby guiding acute and long-term COVID-19 management strategies.

## 2. Materials and Methods

This systematic review was conducted in accordance with the guidelines from the Cochrane Handbook for Systematic Reviews and Preferred Reporting Items for Systematic Reviews and Meta-Analyses (PRISMA). Because of the characteristics of the study topic and the need for timely dissemination of the results, this systematic review and meta-analysis were conducted using a preplanned protocol and registered in PRESPERO later. The protocol is currently under review in PROSPERO.

### 2.1. Search Strategy and Inclusion/Exclusion Criteria

We searched scientific and medical databases (PubMed, Embase, MEDLINE, and Cochrane) for relevant studies published between 1 January 2020 and 30 June 2023. We conducted a database search on 23 July 2023. Our strategy consisted of searching Medical Subject Headings (MeSH) keywords in the databases: long COVID-19 (long COVID, post-acute COVID, and after COVID) and antiviral drugs (remdesivir, nirmatrelvir, and molnupiravir). We included only antiviral agents with proven efficacy against COVID-19 in randomized clinical trials [13,14,15]. Detailed search strategies and procedures are presented in Supplement S1.

The inclusion criteria were as follows: (1) articles reporting clinical results, including the total number of participants and specific number of PASC cases and related deaths/hospitalizations and (2) literature in English. The exclusion criteria were as follows: (1) case reports; (2) no relevant data; (3) gray literature (conference proceedings, dissertations, or theses); (4) studies including children under 18 years of age; and (5) no relevant information on the type of antiviral agents and timing of administration.

### 2.2. Data Extraction and Outcomes

Data extraction was conducted by the reviewers (EJK and YJC) in consultation with the senior author (JYS). The descriptive data extracted in this systematic review included the author, year of publication, study location, diagnostic criteria of COVID-19, participant age, study setting, antiviral agents, and clinical outcomes. We compared the groups that received early antiviral treatment with those who did not. “Early antiviral treatment” means administration within 5 days of the symptom onset following SARS-CoV-2 infection. For each study, we extracted information on population size, age, gender, and follow-up period, as well as details regarding the antiviral type and intervals from symptom onset to administration.

The primary outcome of this study was the rate of PASC occurrence per group (antiviral treatment group vs. control group) and secondary outcomes were PASC-related hospitalization or death. Although the definition of PASC varies, clinical symptoms that persist after COVID-19 include general symptoms (fever, fatigue, malaise), respiratory and cardiac symptoms (cough, shortness of breath, chest pain), neurologic symptoms (difficulty thinking or concentrating, headache, sleeping disorders, anxiety), and gastrointestinal disorders (diarrhea, anorexia). The specific definitions and durations of each study are presented in Table 1.

### 2.3. Risk of Bias Assessment

Two reviewers (EJK and YBS) completed the quality assessments. Disagreements were settled by consensus, and assessments were rendered using the Risk-of-Bias Visualization tool. We evaluated the overall risk of bias using ROBINS-I (Risk Of Bias In Non-randomized Studies—of Interventions) tool. ROBINS-I is composed of seven domains to assess bias due to confounding, selection of participants, intervention classification, deviation from the intended intervention, missing values, measurement of outcomes, and selection of reported results. Each domain of ROBINS-I is rated as ‘yes’, ‘probably yes’, ‘probably not’, ‘no’, or ‘no information’, and the overall risk of bias judgement is categorized into low, moderate, serious, or critical.

### 2.4. Statistical Analysis

To merge effects reported in two or more studies, meta-analyses using a fixed-effects model (Mantel–Haenszel method) were performed with Stata version 10.0 (StataCorp, College Station, TX, USA), thereby estimating the pooled odds ratio (OR). If the results were shown using relative risk (RR) or hazard ratio, we converted the results to OR using the methods defined in the Cochrane Handbook for Systematic Reviews of Interventions [16]. OR with 95% confidence intervals (CIs) are presented. Heterogeneity was assessed using the I^2^ statistic, and a value greater than 50% was considered statistically significant.

To assess for publication bias, we visually inspected a funnel plot and performed Egger’s regression test to verify the presence of publication bias at a significance level of less than 0.05. A threshold of *p* < 0.10 was selected as an indicator of significant publication bias.

## 3. Results

**Figure 1:**
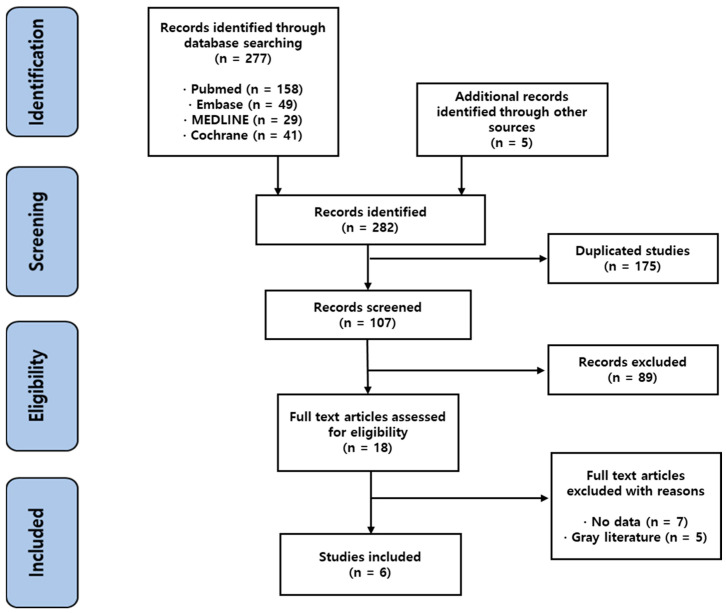
PRISMA flow diagram.

Of the 282 identified studies, 6 were eligible for the analysis, excluding 175 duplicated studies, 89 studies fitting the exclusion criteria, 7 studies without relevant data, and 5 gray literature studies (Figure 1). There were seven papers without appropriate data. Two papers were excluded because the comparison and control groups were not established, and two papers included children and adolescents in the target population. Three papers did not present appropriate OR or RR values that could be used in this study. There were also five gray literature studies, which included short progress reports presented at conferences, which had not been peer-reviewed. Egger’s regression test indicated no significant publication bias (*p =* 0.7218), as shown by the funnel plot (Figure S1 in the Supplementary Materials).

### 3.1. Subsection Characteristics of Included Studies

The specific details of each study are presented in Table 1 [9,17,18,19,20,21]. The studies were conducted in four countries (the Netherlands, Italy, Taiwan, and the United States), with two studies investigating a global health collaborative clinical research platform called TriNetX [18,19] and the other two investigating the US Department of Veterans Affairs healthcare database [20,21]. In total, these studies included a sample size of 3,352,235. Three studies were retrospective cohort studies, and two large-scale studies used 1:1 propensity score matching [18,19]. The number of participants ranged from 649 to 2,361,239, and the mean age ranged from 55.9 (±16.7) to 69.8 (±11.7) years [17,18,19,21]. Most studies involved individuals 18 years of age or older who tested positive for SARS-CoV-2.

All six studies compared patients receiving antiviral treatment with those without antiviral treatment: two studies with nirmatrelvir/ritonavir [18,19,20], one with remdesivir [9], one with molnupiravir [21], and one with any antiviral agent, including nirmatrelvir/ritonavir, molnupiravir, or remdesivir [17].

### 3.2. Risk of Bias

**Figure 2:**
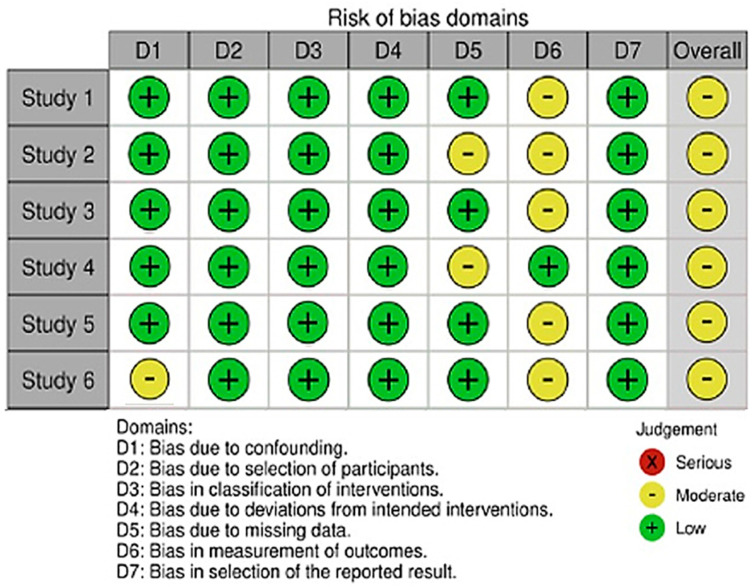
Risk of bias summary for included studies. Study 1: Chilunga et al. [9]; Study 2: Bertuccio et al. [17]; Study 3: Chuang et al. [18]; Study 4: Liu et al. [19]; Study 5: Xie et al. [20]; and Study 6: Xie et al. [21].

With respect to the risk of bias assessment for six studies in this meta-analysis, one study had a moderate risk of bias due to confounding, two studies had a moderate risk of bias due to missing data, and five studies had a moderate risk of bias in the selection of the participants and the reported results. Accordingly, no study was judged to have a ‘high risk of bias’ in the seven domains, and all studies were judged to have a moderate risk of bias. Thus, all studies were included in the analysis (Figure 2).

### 3.3. Effectiveness of Antiviral Therapy on Post-Acute Sequelae of COVID-19

The follow-up period in the six studies ranged from 1 to 12 months [17,19]. The definition of PASC varied considerably among studies but was mostly consistent with the inclusion of any systemic symptoms (cardiovascular, coagulation and hematological, fatigue and malaise, gastrointestinal, kidney, musculoskeletal, metabolic, neurologic, and pulmonary).

Most incidents of PASC were reported by the patients, and most data were collected from electronic medical records. Antiviral administration was performed within 5 days. In these studies, the prevalence of PASC varied from 1.2% to 30.4% [17,19].

**Figure 3:**
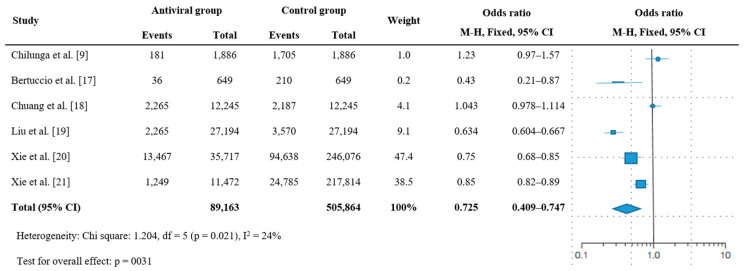
Forest plot for the effectiveness of antiviral therapy on post-acute sequelae of COVID-19. Abbreviations: CI, confidence interval; M–H, Mantel–Haenszel.

The PASC was 27.5% lower in the antiviral treatment group than in the nonantiviral group (k = 6, OR = 0.725, 95% CI = 0.409–0.747, I^2^ = 24%; Figure 3); this difference was significant (*p* = 0.031). A high level of heterogeneity was observed (Q = 1.204, df = 5, *p* = 0.021).

### 3.4. Effectiveness of Antiviral Therapy on Hospitalization or Death

Four studies had data on death and hospitalization [17,18,20,21]. One study was excluded from the analysis owing to the absence of deaths in the antiviral treatment group [17].

**Figure 4:**
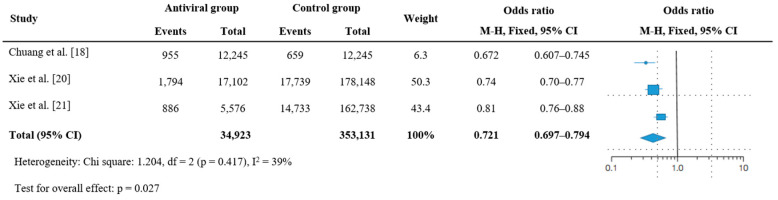
Forest plot for the effectiveness of antiviral therapy on death or hospitalization among patients with post-acute sequelae of COVID-19. Abbreviations: CI, confidence interval; M–H, Mantel–Haenszel.

The remaining three studies reported relevant results [18,20,21]. Hospitalization and emergency room visits were reduced by 67.2% in the nirmatrelvir/ritonavir group (95% CI = 0.607–0.745, *p* < 0.005) [18]. Similarly, the absolute risk reduction at 180 days for hospitalization or death was 2.15 (95% CI = 1.85–2.46) and 2.00 (95% CI = 1.37–2.62) in the nirmatrelvir/ritonavir and molnupiravir groups, respectively [20,21]. The total prevalence of hospitalization or death in the antiviral treatment group was 27.9% lower than that in the nonantiviral treatment group (k = 3, OR = 0.721, 95% CI = 0.697–0.794, I^2^ = 39%, *p* = 0.027; Figure 4). Although a high level of heterogeneity was observed, it was not significant (Q = 0.672, df = 1, *p* = 0.287).

## 4. Discussion

In this study, we analyzed six studies that provided data on PASC and antiviral therapy, including 3,352,235 individuals from different countries. The main finding of this study was that PASC was significantly reduced by 27.5% in patients who received antiviral therapy during early SARS-CoV-2 infection than in those who did not. The risks of PASC-related hospitalization and death were also significantly reduced by 29.7%.

The high PASC-related disease burden is an emerging challenge with respect to national health and economic costs. From the first year of the COVID-19 pandemic in 2020, it was already predicted that long COVID-related medical costs would be significant [7]. Before the COVID-19 pandemic, the prevalence of myalgic encephalomyelitis or chronic fatigue syndrome (ME/CFS) was 1.5 million, with an economic impact of US dollar (USD) 36–51 billion in the US. After the COVID-19 pandemic, the prevalence might rise to USD 5–9 million, causing a potential economic impact of USD 149–362 billion, demanding National Institutes of Health funding to increase to USD 500 million [22,23,24]. Additionally, the burden of lost earnings cannot be ignored. According to a survey of individuals experiencing long COVID, those with significant disabilities may experience a reduction in workforce participation of approximately 70%, resulting in a net income loss of USD 1 trillion [7,13,25]. Economic losses, reduced quality of life, reduced incomes, and increased healthcare expenditures, are estimated to reach USD 3.7 trillion, representing 17% of the GDP in 2019 [7].

Regarding the pathogenesis of long-term COVID, one hypothesis is that symptoms persist due to the long-lasting inflammation caused by the persistence of SARS-CoV-2 in the body [26]. Several studies have observed viral RNA in patient tissues, especially in the intestine, even after long periods of time [27,28]. SARS-CoV-2 first enters the cell via the ACE2 receptor [29], which is associated with various inflammatory conditions, such as multiorgan failure, acute respiratory distress syndrome, and myocardial injury [30]. Thus, persistently present viral antigens result in ongoing inflammation. All antiviral drugs inhibit viral replication, regardless of their mechanism of action; therefore, they are effective in reducing the viral load and improving symptoms associated with COVID-19 [31,32,33,34,35]. To date, three antiviral drugs have proven efficacy in reducing COVID-19 severity in the elderly, and the mechanism of each drug has been elucidated [31,32,36,37]. Remdesivir (Vekluly^®^) was first approved by the Food and Drug Administration (FDA) as an antiviral agent for COVID-19, followed by oral nirmatrelvir/ritonavir (Paxlovid^®^) and molnupiravir (Lagevrio^®^).

Another hypothesis for the pathogenesis of long COVID is the persistence of immune dysregulation. In patients with long COVID, T-cell alterations (depletion of T cells and reduced CD4+ and CD8+ effector memory cell numbers) have been reported to persist for at least 13 months [38,39]. Immune-modulating drugs, such as corticosteroids, which modulate the T-cell response to SARS-CoV-2 infection, can reduce the number of PASCs; therefore, additional research is required regarding the correlation between these drugs and PASC.

This study had some limitations. First, all the included studies were observational in design, which had inherent limitations, such as selection bias and residual confounding, thereby affecting the results. Additionally, as four studies used the healthcare system databases TriNetX [18,19] and Veterans Affairs [20,21], there may be misdiagnoses, inaccurate coding, and missing data. Second, the number of groups was small and the heterogeneity among groups was high. The number of enrolled patients varied for each study, and there were significantly more males than females in two of the studies [20,21]. There were also differences in the follow-up period and type of antiviral agent administered. In a previous randomized controlled study (SOLIDARITY Finnish Clinical Trial) for remdesivir, there was no difference in symptom improvement after 1 year between the remdesivir-treated group (85%) and standard treatment group (86%) [40]. This study evaluated several types of antiviral drugs at once; however, further studies evaluating individual antiviral drugs are needed. Finally, the symptoms of PACS were subjectively expressed by patients in most studies. If an objective index to evaluate long-COVID-related symptoms is established, additional studies are warranted to qualitatively and quantitatively evaluate the effect of antivirals.

## 5. Conclusions

To the best of our knowledge, this is the first systematic review and meta-analysis to evaluate the effectiveness of antivirals on long COVID and related morbidity/mortality. This meta-analysis showed that early antiviral treatment is associated with a reduced risk of PASC, hospitalizations, and deaths due to long COVID. Thus, early antiviral therapy is recommended for older adults and individuals with at-risk conditions to reduce the risk and severity of long COVID.

## Figures and Tables

**Table 1:** Characteristics of the included studies.

Study	Design	Population	No.	Male (%)	Median (Range) or Mean (SD) Age	Nation	Follow-Up Duration	PASC		Antiviral Therapy			Death or Hospitalization
								Definition	No.	Drug	No. (%)	Intervals from Diagnosis to Treatment	
Chilunga et al. [9]	Retrospective cohort	≥18 years old, either a confirmed positive SARS-CoV-2 PCR or high clinical suspicion for COVID-19 based on clinical presentation and computed tomography imaging	1886	56.5	62 (59–71)	The Netherlands	12 weeks	NICE guideline [2]	483	Remdesivir	181 (9.6)	-	.
Bertuccio et al. [17]	Retrospective observational	>18 years old with positive SARS-CoV-2 nasopharyngeal swab, mild/moderate symptoms, at least one risk factor for COVID-19 progression	649	51.6	67 (54–76)	Italy	1/3 months	U.S. CDC COVID-19 symptom list [a]	323	Nirmatrelvir/ritonavir, molnupiravir, and remdesivir	197 (30.4)	-	Death or hospitalization
Chuang et al. [18]	Retrospective cohort	≥18 years old with positive test for SARS-CoV-2 or received a diagnosis of COVID-19	477,382	42.7 *	55.9 (16.7) *	Taiwan	90 to 180 days	Fatigue, pain, dizziness, headache, cognitive impairment, cardiopulmonary symptoms, abdominal symptoms, anxiety or depression, and sleep disorders	4452	Nirmatrelvir/ritonavir	12,245 (2.6)	Within 5 days	Hospitalization or emergency room visits
Liu et al. [19]	Retrospective cohort	≥18 years old with positive test for SARS-CoV-2 or received a diagnosis of COVID-19, at high risk of severe COVID-19	2,361,239	41.7 *	59.4 (15.0) *	Taiwan	90 days to 1 year	Neuropsychiatric sequela	5835	Nirmatrelvir/ritonavir	27,194 (1.2)	-	.
Xie et al. [20]	Observational cohort	With positive SARS-CoV-2 test, at least one risk factor of progression to severe acute COVID-19 illness	281,793	87.9	65.7 (13.4)	U.S.	180 days	Incident ischemic heart disease, dysrhythmia, DVT, PE, fatigue/malaise, liver disease, acute kidney injury, muscle pain, diabetes, neurocognitive impairment, dysautonomia, shortness of breath, cough	40,098	Nirmatrelvir/ritonavir	35,717 (12.7)	Within 5 days	Death or hospitalization
Xie et al. [21]	Observational cohort	Same as above	229,286	91.6	69.8 (11.7)	U.S.	180 days	Incident ischemic heart disease, dysrhythmia, DVT, PE, fatigue/malaise, liver disease, acute kidney injury, muscle pain, diabetes, neurocognitive impairment, dysautonomia, shortness of breath, cough	29,743	Molnupiravir	13,007 (5.7)	Within 5 days	Death or hospitalization

* Data after matching. Abbreviations: PCR, polymerase chain reaction; NICE, National Institute for Health and Care Excellence; PASC, post-acute sequelae of COVID-19.

## Data Availability

The data presented in this study are available on request from the corresponding author.

## Supplementary Materials

The following supporting information can be downloaded at https://www.mdpi.com/article/10.3390/jcm12237375/s1, Word Search Strategy; Figure S1: Funnel plot for included studies.
