# Supplementary material for: Effectiveness of Antiviral Therapy on Long COVID: A Systematic Review and Meta-Analysis
Source: J Clin Med. 2023 Nov 28;12(23):7375. doi: 10.3390/jcm12237375 (PMC10707593; doi:10.3390/jcm12237375)

Word Search Strategy

1. Long COVID.mp. [mp=title, abstract, heading word, drug trade name, original title, device manufacturer, drug manufacturer, device trade name, keyword, floating subheading word, candidate term word]

2. Post-acute COVID.mp. [mp=title, abstract, heading word, drug trade name, original title, device manufacturer, drug manufacturer, device trade name, keyword, floating subheading word, candidate term word]

3. After COVID.mp. [mp=title, abstract, heading word, drug trade name, original title, device manufacturer, drug manufacturer, device trade name, keyword, floating subheading word, candidate term word]

4. 1 or 2 or 3

5. remdesivir.mp. [mp=title, abstract, heading word, drug trade name, original title, device manufacturer, drug manufacturer, device trade name, keyword, floating subheading word, candidate term word]

6. nirmatrelvir.mp. [mp=title, abstract, heading word, drug trade name, original title, device manufacturer, drug manufacturer, device trade name, keyword, floating subheading word, candidate term word]

7. molnupiravir.mp. [mp=title, abstract, heading word, drug trade name, original title, device manufacturer, drug manufacturer, device trade name, keyword, floating subheading word, candidate term word]

8. antiviral*.mp. [mp=title, abstract, heading word, drug trade name, original title, device manufacturer, drug manufacturer, device trade name, keyword, floating subheading word, candidate term word]

9. 5 or 6 or 7 or 8

10. 4 and 9

Figure S1. Funnel plot for included studies

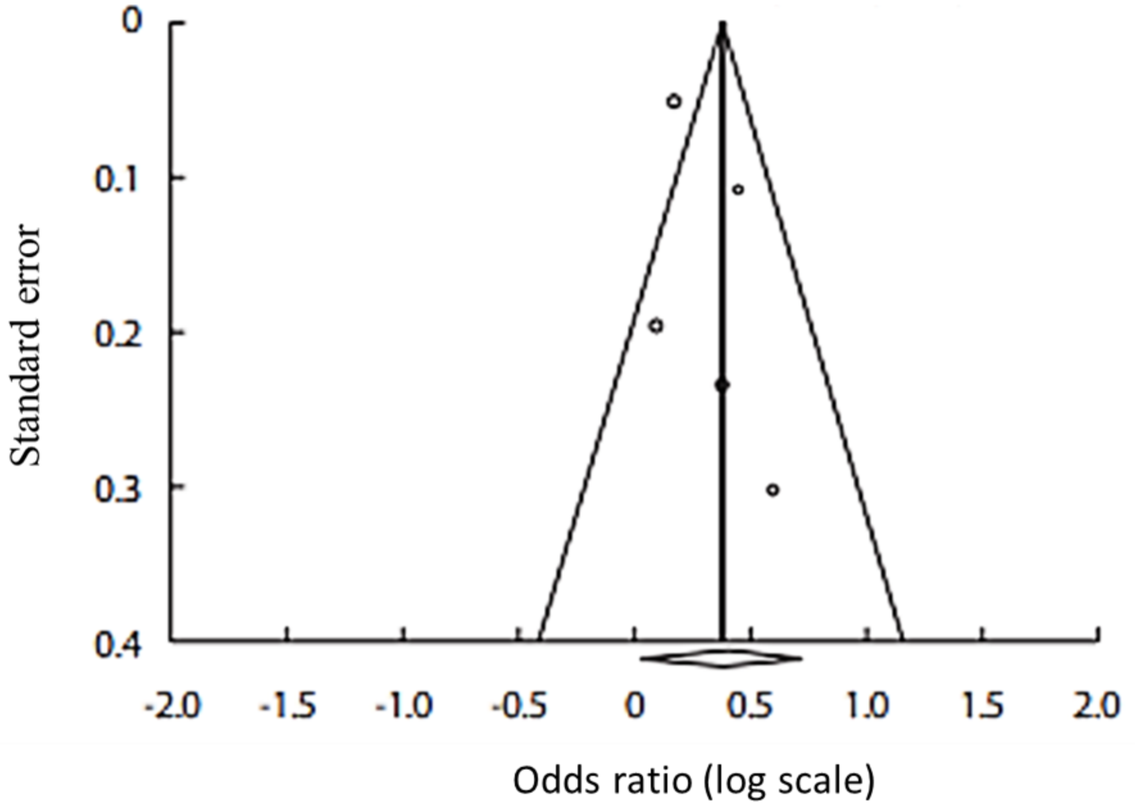

Supplement: Supplementary file 1 [file jcm-12-07375-s001.zip › jcm-2642118-supplementary.pdf]
